# Estimating the location of baleen whale calls using dual streamers to support mitigation procedures in seismic reflection surveys

**DOI:** 10.1371/journal.pone.0171115

**Published:** 2017-02-15

**Authors:** Shima H. Abadi, Maya Tolstoy, William S. D. Wilcock

**Affiliations:** 1 School of STEM, University of Washington, Bothell, WA, United States of America; 2 Lamont–Doherty Earth Observatory, Columbia University, Palisades, NY, United States of America; 3 School of Oceanography, University of Washington, Seattle, WA, United States of America; Institute of Deep-sea Science and Engineering, Chinese Academy of Sciences, CHINA

## Abstract

In order to mitigate against possible impacts of seismic surveys on baleen whales it is important to know as much as possible about the presence of whales within the vicinity of seismic operations. This study expands on previous work that analyzes single seismic streamer data to locate nearby calling baleen whales with a grid search method that utilizes the propagation angles and relative arrival times of received signals along the streamer. Three dimensional seismic reflection surveys use multiple towed hydrophone arrays for imaging the structure beneath the seafloor, providing an opportunity to significantly improve the uncertainty associated with streamer-generated call locations. All seismic surveys utilizing airguns conduct visual marine mammal monitoring surveys concurrent with the experiment, with powering-down of seismic source if a marine mammal is observed within the exposure zone. This study utilizes data from power-down periods of a seismic experiment conducted with two 8-km long seismic hydrophone arrays by the R/V *Marcus G*. *Langseth* near Alaska in summer 2011. Simulated and experiment data demonstrate that a single streamer can be utilized to resolve left-right ambiguity because the streamer is rarely perfectly straight in a field setting, but dual streamers provides significantly improved locations. Both methods represent a dramatic improvement over the existing Passive Acoustic Monitoring (PAM) system for detecting low frequency baleen whale calls, with ~60 calls detected utilizing the seismic streamers, zero of which were detected using the current *R/V Langseth* PAM system. Furthermore, this method has the potential to be utilized not only for improving mitigation processes, but also for studying baleen whale behavior within the vicinity of seismic operations.

## I. Introduction

Marine mammals use sound for their important life functions such as communicating, navigating, and finding food or a mate. Ocean noise pollution has increased greatly in recent years due to human activities in the ocean [1]. Seismic surveys are one of the more common high source-level anthropogenic sounds in the ocean. The high sound intensity of the airguns involved in seismic surveys has led to concerns over their effects on marine life [2]. Since seismic surveys use low frequency sound to image structure beneath the seafloor, the potential impact on baleen whales that communicate in the same frequency range may be particularly significant. It has been shown that bowhead whales exposed to seismic sources interrupt their normal activities and move away [3]. One study shows that bowhead whale calling rates near seismic operations increase initially at cumulative sound exposure level of ~94 dB re 1 μPa^2^-s, but then begin decreasing for values above ~127 dB re 1 μPa^2^-s until ~160 dB re 1 μPa^2^-s when whales are silent [4]. Avoidance behavior has also been seen in humpback whales [5] and possibly in blue whales [6].

To better mitigate against potential impacts of seismic sources, the acoustic received levels from the U.S. academic community’s seismic vessel, the *R/V Marcus G*. *Langseth*, have been measured to quantify the exposure radii, based on the criteria defined by the National Marine Fisheries Service [7, 8, 9, 10]. To make sure marine mammals do not enter the exposure radii, experienced marine mammal observers monitor the area visually and acoustically with a short hydrophone array that can detect, but not locate, some marine mammal calls. While the technique is inherently limited to animals that are calling, the ability to locate marine mammal calls during seismic surveys could help demonstrate the effectiveness of the observations and the mitigation process and might add an additional level of safety to existing monitoring methods.

Several sound source localization techniques have been developed to locate and track marine mammals using different types of receiving arrays such as vertical arrays [11, 12], networks of seafloor receivers [5, 13], and towed horizontal arrays [14–18]. Towed horizontal arrays in particular, have been used extensively in marine mammal localizations. For instance: Tran et al., [17] used the moving towed array triangulation technique for estimating the range of sperm whales; Barlow and Taylor [16] used a short towed array to estimate sperm whale abundance from a combined acoustic and visual survey; Thode [14] used the relative arrival times between the direct and the surface reflected paths, measured by short horizontal arrays, to track sperm whales and extended this approach to account for the refraction of rays arising from a depth-dependent sound speed [15]; and Gong et al., [18] applied the array invariant method [19] to understand the effect of sonar on humpback calls.

The method used in this study, the travel time residual method (TTR) [20], utilizes the full capabilities of long seismic streamers to estimate the horizontal location of low-frequency sound sources. Beamforming is used to estimate the angle of arriving energy relative to sub-arrays of the streamer, which constrains the horizontal propagation velocity to each sub-array for a given trial location. A grid search method is then used to minimize the time residual for relative arrival times along the streamer estimated by cross correlation. This method was presented in a previous study [20] and used to find the location of low frequency sound sources (specifically airgun signals) using a single streamer during turns between track lines in a seismic survey off the coast of Washington state. In this paper, we extend the method to the use of dual streamers and we use it to locate low-frequency baleen whale calls during a seismic reflection survey off Alaska in order to assess the effectiveness of the mitigation procedures.

The remainder of this paper is divided into six sections as follows: Section II describes the seismic experiment, the source and receiver equipment, and the associated monitoring surveys (visual and acoustic); Section III presents a brief overview of the mathematical formulation of the sound source localization technique; Section IV presents the sound source location results from a simulated normal mode propagation in a simple environment that mimics the seismic experiment, to elucidate the left-right ambiguity and the source-to-array orientation; Section V presents the whale call location results during the mitigation procedures; Section VI discusses the results; and Section VII summarizes the conclusions of the study.

## II. Alaska 2011 experiment

**Fig 1:**
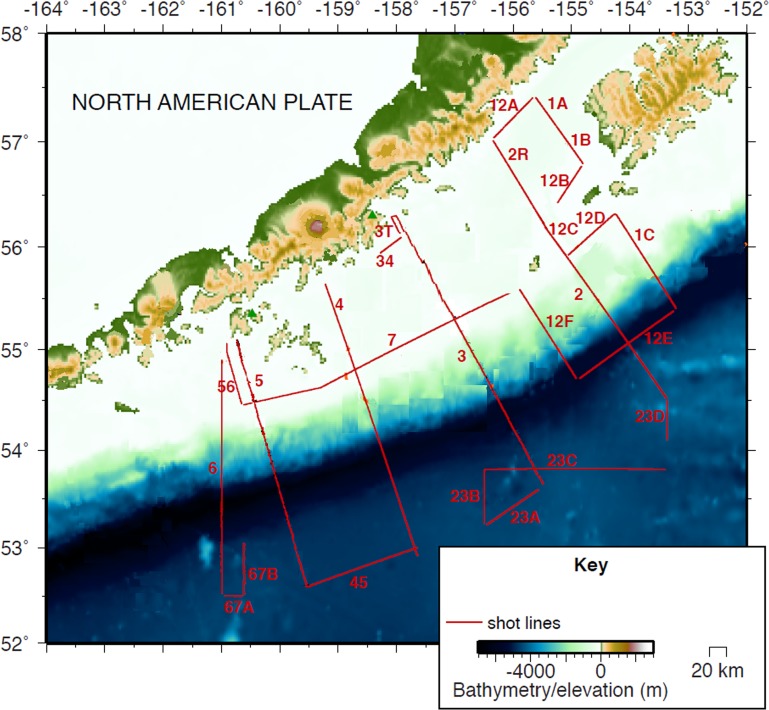
Map of the ALEUT cruise track lines. Adapted from Shillington et al., (2015), map shows the track lines of the two-streamer MCS data collected during the 2011 ALEUT experiment. Shots were fired every 62.5 m, except during periods of shut-down due to marine mammal mitigation.

The data utilized in this paper are from the Alaska Langseth Experiment to Understand the megaThrust (ALEUT) seismic reflection experiment [21] conducted by the *R/V Marcus G*. *Langseth* on cruise MGL1110, from 11 July 2011 until 5 August 2011. The aim of this cruise was to characterize the megathrust, the overriding and down-going plates, and other fault systems associated with the Alaska-Aleutian subduction zone. The survey plan included multi-channel seismic (MCS) survey lines, which were oriented both north to south and east to west in a wide range of water depths from <25 meters to >6000 m (Fig 1). Survey lines varied in length from approximately 20 to 400 kilometers.

### A. Equipment

**Fig 2:**
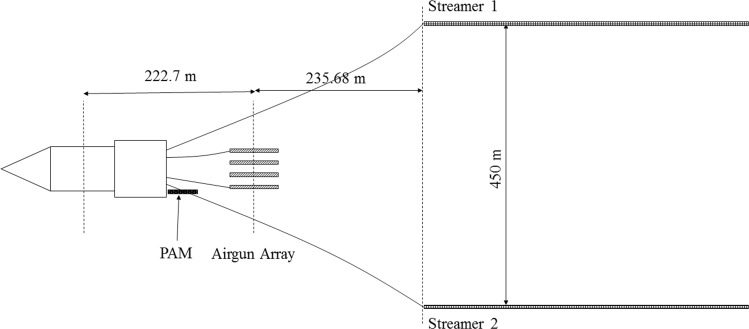
R/V *Langseth* towing configuration. Data is acquired using two seismic streamers which are 8-km long and comprised of 636 hydrophone channels.

Data were acquired using a four-string airgun array with a volume of 6600 cubic inches and two seismic streamers (Fig 2). Each seismic streamer was 8-km long and comprised of 636 hydrophone channels that were spaced 12.5 m apart. The starboard streamer (streamer 1) and the port streamer (streamer 2), were towed from paravanes with a nominal separation of 450 m. The airgun array was towed 223 m behind the ship’s navigational reference point, the center of the vessel (Fig 2). The distance from the center of the source to each streamer was 236 m. The source array and one of the streamers were towed at a depth of 12 m while the second streamer was towed at 9 m.

The airgun shots occurred every 62.5 m while the ship moved at a speed of ~4.5 knots, equivalent to a shooting interval of ~27 s. After each shot, streamer data was recorded for 22.5 s with a sampling rate of 500 Hz following the application of a 220-Hz low-pass anti-alias filter.

The airgun and individual hydrophone positions were determined using differential GPS observations between the vessel, the seismic source array, and a streamer tailbuoy, together with compass headings on the streamer, and range and bearing data from transducers on the source, streamer, and tailbuoy. The vessel position is known with sub-meter accuracy and the position error on the streamer varies from 1.5 m to 4 m going from near to far hydrophone channels.

### B. Marine mammal monitoring surveys

All *R/V Langseth* cruises utilizing airguns conduct marine mammal monitoring surveys concurrent with the seismic experiment. The monitoring survey uses a combination of visual and acoustic watches to minimize potential impacts on marine mammals. The visual monitoring is carried out from an observation tower located 19 meters above the water surface, which affords the Protected Species Observers (PSOs) a 360-degree viewpoint around the acoustic source. In addition to the visual monitoring, a passive acoustics monitoring (PAM) system is used to monitor marine mammal calls in the survey area. The PAM system consists of a short hydrophone array towed behind the research vessel with four elements: three broadband hydrophones (2 kHz to 200 kHz) and one lower frequency hydrophone (75 Hz to 30 kHz).

### C. Mitigation procedure

The exposure zone is the area where 180 and 190 dB re 1μPa sound levels are received and is defined for different species at various water depths by National Marine Fisheries Services (NMFS) as shown in Table 1. When a marine mammal is observed within the exposure zone calculated for a 4-string airgun array (6,600 cubic inch), the seismic source is powered down to the mitigation gun (40 cubic inch airgun) to avoid unnecessary disturbance to the marine environment. If the animal is in the exposure zone calculated for a single airgun, the seismic source is shut down completely. In order to resume full volume shooting, a “ramp-up” or “soft-start” of the airgun array is mandatory. During this process, the number of airguns firing increases to warn marine mammals in the vicinity and to provide enough time for them to leave the area and thus avoid any potential injury or impairment of their hearing. The ramp-up procedure can be started if no marine mammal is observed in the exposure zone for at least 30 minutes [22]. All the whale calls used in this study were recorded during the mitigation procedure.

**Table 1:** Safety radii used in the ALEUT survey program.

*Source & Volume*	*Water Depth (m)*	*Predicted RMS Distances (m)*	
		*190 dB (Pinnipeds)*	*180 dB (Cetaceans)*
*Single Airgun (40 in*^*3*^*)*	>1000	12	40
	100–1000	18	60
	<100	150	296
*4 Strings*, *36 Airgun Source (6*,*600 in*^*3*^*)*	>1000	460	1100
	100–1000	615	1810
	<100	770	2520

Safety radii define the exposure zones and are the predicted distances at which 180 and 190 dB re 1μPa sound levels are received.

### III. Sound source localization technique using the seismic streamer

The travel time residual (TTR) method [20] is a grid-search technique that estimates the two-dimensional horizontal location of a sound source using data recorded by seismic streamers. Each streamer is divided into *M* sub-arrays, each comprising *N* hydrophones. Delay and sum beamforming is applied to each sub-array to calculate the propagation angle of the received signal relative to the streamer (this angle defines a cone around the streamer). For a given trial source location, this beamformer angle can be used to determine either that the location is infeasible because the location lies outside the beamforming cone, or to compute the horizontal component of the wave propagation velocity (the average group velocity of all the propagating modes). For feasible locations, the travel time between an element in the *j*^*th*^ sub-array and the potential source location in the search grid, $t_{j}^{c}$, can be calculated simply by dividing the distance by horizontal velocity obtained for that location. Additionally, the relative observed time at that element, $t_{j}^{o}$, can be calculated by the maximum cross correlation coefficient between the signals received by that element and a reference element which is fixed for all sub-arrays. Here, the furthest hydrophone from the airgun array is chosen as the reference element to minimize the influence of noise. At the end, the travel time residual, *R*_*t*_(*x*,*y*), between $t_{j}^{o}$ and $t_{j}^{c}$ is calculated for all points in the search grid that are feasible source locations for each sub-array, according to

$$R_{t}(x,y)=\sqrt{\frac{1}{M}\sum_{j=1}^{M}{\left(t_{j}^{o}-T_{0}-t_{j}^{c}\right)}^{2}}$$ (1)

where *T*_*0*_ is the origin time of the sound relative to the time the signal was received at the reference element, and can be estimated by

$$T_{0}=\frac{1}{M}\sum_{j=1}^{M}\left(t_{j}^{o}-t_{j}^{c}\right)$$ (2)

**Fig 3:**
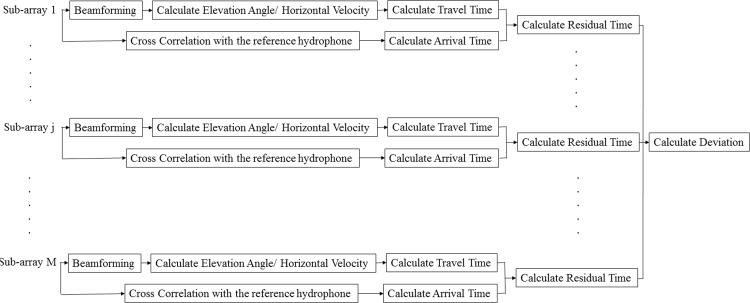
A block-diagram description of travel time residual localization method.

The estimated source location is the grid point where *R*_*t*_(*x*,*y*) is minimized. A simple block-diagram description of the TTR method is shown in Fig 3.

## IV. Simulation results

**Fig 4:**
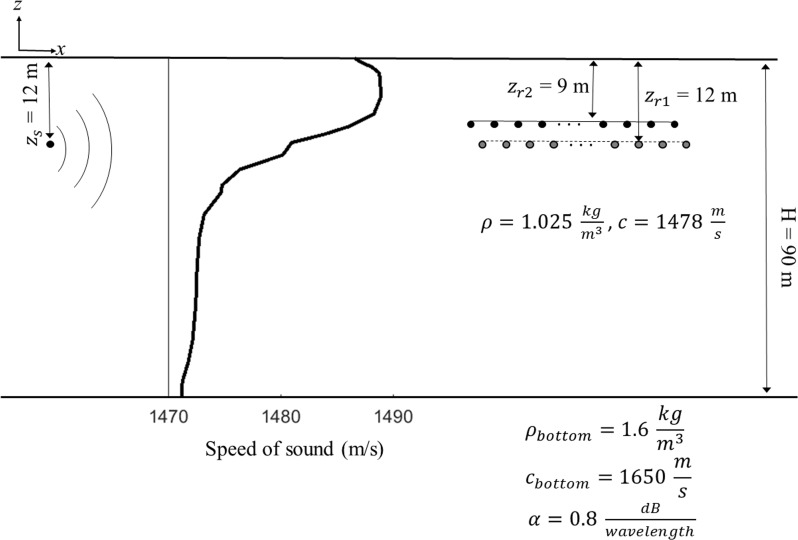
Array geometry and range-independent two-layer waveguide model used in simulations that mimic the experiment. The sound speed profile and the average water density used in this model are taken from an XCTD measurement on July 29^th^.

To assess the performance of the sound source localization technique, we consider a 90-m-deep water channel that mimics the shallow water environment of Alaska. The normal mode propagation algorithm *KRAKEN* [23] is used to propagate an 800 ms chirp through a simple two-layer waveguide shown in Fig 4. Different bandwidths are selected to mimic the whale calls we discuss below in Section V.

The simulation presented in this section is conducted to answer two questions: 1) Can the travel time residual method resolve left-right ambiguity and thus be used once the streamers are straight? 2) What is the impact of noise, signal bandwidth, and source-to-array range and orientation on the estimated locations?

Previously, the TTR method was used to estimate sound source locations using data recorded by a single streamer on turns between track lines [20]. For monitoring purposes, it is also important to find the range of the calling animals when the streamer is being towed along straight track lines. When the recording array is straight, resolving left/right ambiguity is always a challenge. However, it is almost impossible to deploy a perfectly straight streamer in real surveys. Here, simulations are undertaken to compare the locations between a perfectly straight streamer and a typical example of the actual streamer configuration during the Alaska experiment which has ~250 m deviation from the best fitted straight line. The location results using dual streamers are also presented to show how the use of the second streamer increases the resolution.

**Fig 5:**
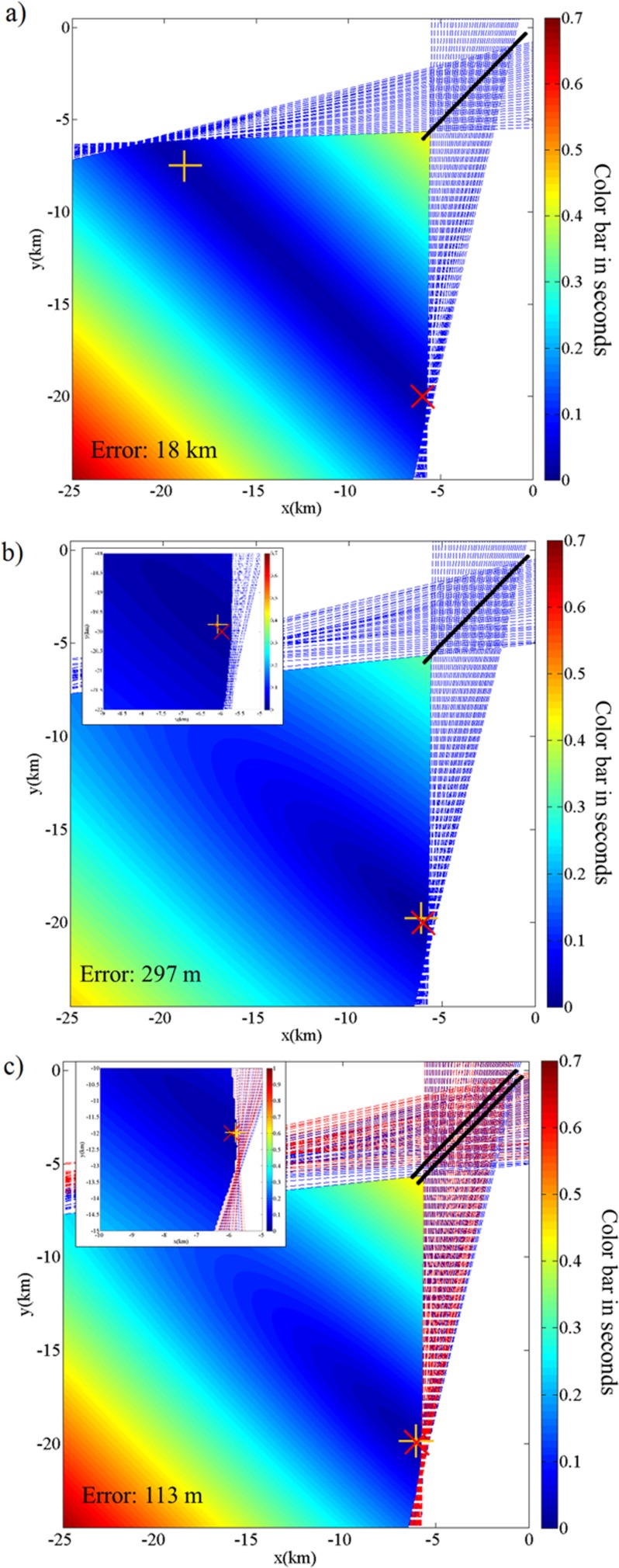
Simulation location results using different streamer configurations. (a) single straight streamer, (b) actual single streamer, (c) actual dual streamers. The dashed lines are the lateral edges of the beamformer cone (see [20]), for each selected sub-array. The color shading shows the travel time residual (in seconds) from Eq 1. The solid black line shows the location of the streamer relative to the center of the airgun array. The actual source location and the estimated source location are shown by a red cross and a yellow plus symbol, respectively.

Fig 5 shows the simulated source location results using a single straight streamer (part a), the actual single streamer used in the Alaska experiment (part b), and the actual dual streamers used in the Alaska experiment (part c). The source location result using a single straight streamer is symmetric and the choice between the left and right side is a result of grid resolution and rounding error. The location error is defined as the absolute distance between the true and the estimated source locations. In this example, the actual single streamer resolves the left-right ambiguity and adding the second streamer reduces the location error by ~60%.

**Fig 6:**
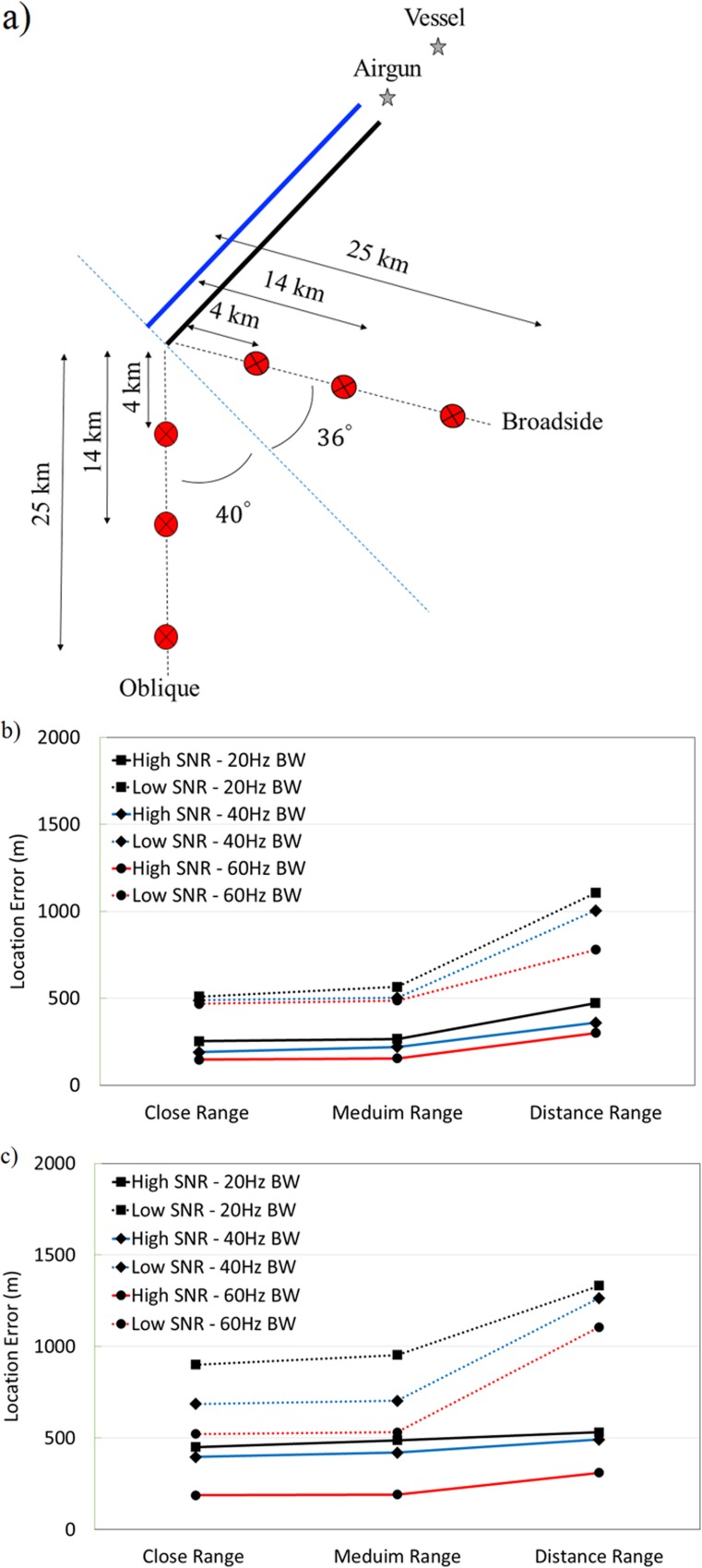
Simulation location error study. (a) Source-to-array configurations used in the simulations of broadside and oblique sources, (b) Broadside location error, (c) Oblique location error. The solid lines and the dashed lines show the high and low SNR results, respectively.

Fig 6B and 6C show the effects of the source-to-array orientation, range, SNR, and bandwidth on the location error using the dual streamers. Two array configurations are considered (Fig 6A): an oblique source behind the streamers on the starboard side and a broadside source also on the starboard side. For each array configuration, three source-to-array ranges (the distance between the sound source and the far end of the starboard streamer) are considered: a close range of 4 km; a medium range of 14 km; and a distant range of 25 km. In each geometry, three signal bandwidths (20, 40, and 60 Hz) are analyzed. The center frequency is kept constant at the streamer design frequency which is 60 Hz. White noise has been added to the simulation to generate variable SNRs along the streamer; lower SNR at the elements close to the vessel and higher SNR at the elements far from the vessel. The averaged SNR is ~40 dB for the high SNR results and ~5 dB for the low SNR results. The location error increases with distance to the source and are on average about 40% higher for the oblique source. Low SNR has larger impacts on the errors for the distant sources.

## V. Experimental results

The visual monitoring effort produced a total of 52 baleen whale detections while data was being recorded from the streamers. The focus of this study is on a subset of 25 baleen whale detections (Table 2) where the visual monitoring led to a mitigation action: powering down to the mitigation gun or completely powering off. The R/V *Langseth* PAM short array did not detect calls from any of the observed animals. It is likely that interference from low frequency ship noise limits the sensitivity of the PAM array for detecting baleen whale calls. To search for baleen whale calls in the streamer data, spectrograms were visually inspected for several elements at different positions along each streamer, for all the time periods that PSO’s reported whale sighting in their visual observations report and the airguns were either powered down or shut down. Table 2 summarizes the results of these efforts; calls were detected by the streamers for 40% of the visual detections indicating that individual elements in the seismic streamers are more sensitive than the PAM array, presumably because they are towed further away from the ship (Fig 2). Moreover, some of the calls recorded by the streamers have frequencies below the cutoff frequency of the PAM hydrophones.

**Table 2:** Number of detected baleen whales by visual monitoring, R/V *Langseth* PAM array, and streamer array.

*Detected Species*	*Visual Monitoring*	*Acoustic Monitoring (PAM)*	*Acoustic Monitoring (Streamers)*
Fin Whale	6	0	5
Humpback Whale	14	0	4
North Pacific Right Whale	1	0	0
Unidentified Whale	4	0	1
Total	25	0	10

To study the effectiveness of the mitigation and ramp-up processes, the travel time residual method was applied to the baleen whale calls detected with the seismic streamers. Here, we present the results for four sequences of detections linked to sightings as summarized in Table 3.

**Table 3:** Summary of all the acoustic detections using the streamers data.

*Sighting*	*Date*	*Time duration (UTC) of the acoustically detected calls*	*Water depth (m)*	*Whale call bandwidth (Hz)*	*Number of recorded calls*	*Airgun activity*	*Average SNR (dB)*	*Min/Max acoustically Estimated Range (km)*
*# 1*	July 23, 2011	18:20–18:41	4635–4649	15–40	21	Not Firing-Ramp Up	3.9	1.8/11
*# 2*	July 27, 2011	15:51–16:15	237–222	40–100	14	Not Firing-Ramp Up	4.7	3.6/5.8
*# 3*	July 29, 2011	02:49–02:50 and 05:06–05:23	90–93	40–100	9	Mitigation	3.2	6.2/14.4
*# 4*	July 31, 2011	18:35–19:59	95–85	30–80	15	Not Firing-Ramp Up	4.9	1.6/13.0

## 1. Detection event 1

The first event occurred on July 23, 2011 and comprised a prolonged sighting of a pair of fin whales followed by the detection of the extremely rare north pacific right whale and a humpback whale (Table 4). The pair of fin whales observed at 15:43 was initially sighted traveling antiparallel to the vessel while on a survey line with the source firing on full power. A power-down was implemented and shortly after the whales changed course to approach the vessel, following alongside the vessel, crossing back and forth under the vessel from side to side and approaching as close as 40 m to the side of the ship. The pair remained in the area of the vessel for over three hours. The north pacific right whale was observed at 17:23 blowing close to the airgun and triggered a complete shutdown. The north pacific right whale traveled parallel to the vessel for 47 minutes during which time several blows were observed in addition to the animal fluking and diving twice and pectoral fin slapping once. Over an hour later, a humpback whale appeared under the port streamer.

**Table 4:** Summary of the acoustic and visual detections on July 23, 2011.

*Time of the important events*	*Information from the acoustic recordings*	*Information from the visual observation*	*Connecting the visual and acoustic monitoring*
15:43	Airgun array is firing at full power and the streamer data are too noisy to detect any call. Airgun array is powered down to the mitigation gun at 15:48.	Two fin whales are sighted at 1.5 km slightly outside the exposure zone off the starboard bow travelling toward the vessel at a moderate pace. 3 minutes later, they entered the exposure zone. They were sighted very close to the airgun (~180 m) at 16:07 and 16:36.	Animals are in the exposure zone. Streamer data are too noisy. No connection is found.
17:23	Airgun array is powered off at this time. No call is recorded.	The fin whales left the exposure area from the astern of the airgun. A single north pacific right whale is observed blowing 1 km off the starboard stern heading toward the vessel.	No call is recorded. No connection is found.
18:20	No gun is fired. The first call is recorded by the streamer. The estimated range is 1.8 km.	The fin whales are still being observed outside of the exposure zone until 18:53. Their exact locations are not documented. The north pacific right whale is last sighted blowing 600 m off the starboard stern at 18:10.	The recorded calls are similar to fin whale summer calls [25, 26]. Based on the visual observation, the fin whales are still being observed outside of the exposure zone which confirms the estimated location.
18:32	No gun is fired until 18:40 when the ramp up starts. Whale calls are still being detected on the streamers data. The estimated range is ~11.5 km.	A single humpback is observed surfing just under the port streamer lead, 200 m off the stern. This animal is last sighted at 850 m off the port stern swimming away from the ship at 18:40.	The recorded calls are most likely from the same pair of fin whales that were observed earlier. The lack of visual observations is consistent with their estimated distance from the vessel.

**Fig 7:**
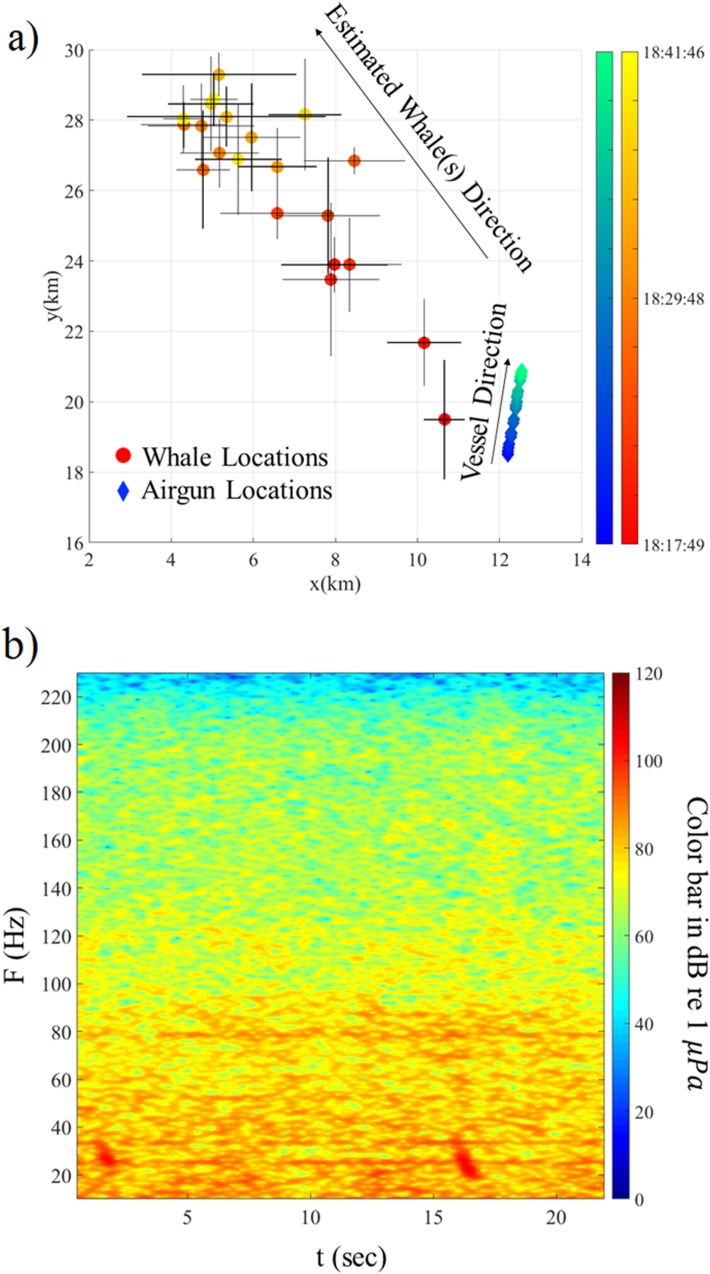
Location results on July 23, 2011. (a) Spatial distribution in Universal Transverse Mercator (UTM) coordinates of the calls recorded on July 23, 2011 relative to a reference point located in zone 5 at (-141, 5955) km. The estimated locations using the travel time residual technique are shown by circles, color coded in red to yellow by time of day (labelled in hours:minutes:seconds in universal time) (early to late). The airgun locations when calls were recorded by the streamer are shown by diamonds color coded in blue to green by time (early to late). The location uncertainties are shown by black lines at the estimated locations. (b) Spectrogram in units of decibels (dB) re 1 μ*Pa* of two example whale calls (visible at ~1 s and ~16 s and 20–40 Hz) recorded on July 23, 2011 at 18:20.

The streamers recorded 21 whale calls in the frequency band 15–40 Hz, well below the PAM detection range, between 18:20 and 18:41 when no gun was firing, except for the last three calls which occur after the ramp-up procedure was started, when we estimate that the whales were at a distance of ~10 km from the airgun array. The water depth beneath the ship varied from 4635 m to 4649 m. Fig 7A shows the spatial distribution in meter coordinates of the recorded calls and also the contemporaneous locations of the airgun array. All the recorded calls have similar spectrograms; an example is shown in Fig 7B.

The recorded calls cannot be linked directly to a contemporaneous sighting. Thus, both the type and the number of calling animals are unknown although the similarity of the spectrograms suggests that all the recordings are from a single species. As shown in Fig 7A, the estimated locations move rapidly away from the airgun array in a northwest direction (~11 km in ~22 minutes). It is unlikely that the north pacific right whales can travel this fast [24]. The call spectrogram (Fig 7B) are consistent with the fin whale summer call [25, 26] and fin whales are recognized as the fastest of the large whales with the maximum speed of 10 m/s [27]. It is thus, possible that the recorded calls are from the pair of fin whales initially observed at 15:43. It was documented that they left the exposure zone at 17:23 and they continued to be observed outside of the exposure zone until 18:53 although their precise range and bearing was not recorded.

### 2. Detection event 2

The second event (Table 5) occurred on July 27, 2011 and included sighting of 6 unidentified baleen whales at 15:11 followed by the detection of 2 unidentified baleen whales at 16:48, 2 humpback whales at 17:21 and one fin whale at 19:06. According to the Protected Species Monitoring Report, these baleen whales were observed from too great a distance, or too briefly, to make a species identification.

**Table 5:** Summary of the acoustic and visual detections on July 27, 2011.

*Time of the important events*	*Information from the acoustic recordings*	*Information from the visual observation*	*Connecting the visual and acoustic monitoring*
15:11	Full power airgun is firing and the streamer data are too noisy to detect any call.	One baleen whale is sighted blowing ~5.8 km off the port bow. Two more baleen whales are sighted off the starboard side. Observers were not able to determine the species.	Streamer data are too noisy. No connection is found.
15:51	The airgun array is powered down at 15:50. The first whale call is detected on the streamer data. The estimated range is 5.6 km off the starboard quarter.	The two whales observed off the starboard are travelling in the opposite direction to the vessel angled slightly away from the vessel heading south.	The observations confirm the acoustic location results. The estimated locations are off the starboard and the first three estimated locations confirm that the animals are swimming in the opposite direction to the vessel.
16:01	A call is recorded 3.5 km off the starboard quarter at 15:59. Another call is recorded 4.5 km astern at 16:15. The ramp-up procedure starts at 16:02 and no more calls are detected on the streamer after that. The ramp-up completes at 16:22.	The animals are sighted slightly astern of the ship outside the exposure area. Three more blows are sighted belonging to three additional whales, all ~4 km away.	The observations confirm the acoustic location results. Both methods show that the animals were at the same distance off the stern.
16:48	Airgun array is firing at full power and the streamer data are too noisy to detect calls.	Two baleen whales are briefly sighted ~4 km away.	Streamer data are too noisy. No connection is found.
17:21	Airgun array is firing at full power and the streamer data are too noisy to detect calls.	Two humpback whales are sighted off the starboard beam heading south.	Streamer data are too noisy. No connection is found.
19:06	Airgun array is firing at full power and the streamer data are too noisy to detect calls.	A fin whale is sighted ~6 km away off the port beam.	Streamer data are too noisy. No connection is found.

**Fig 8:**
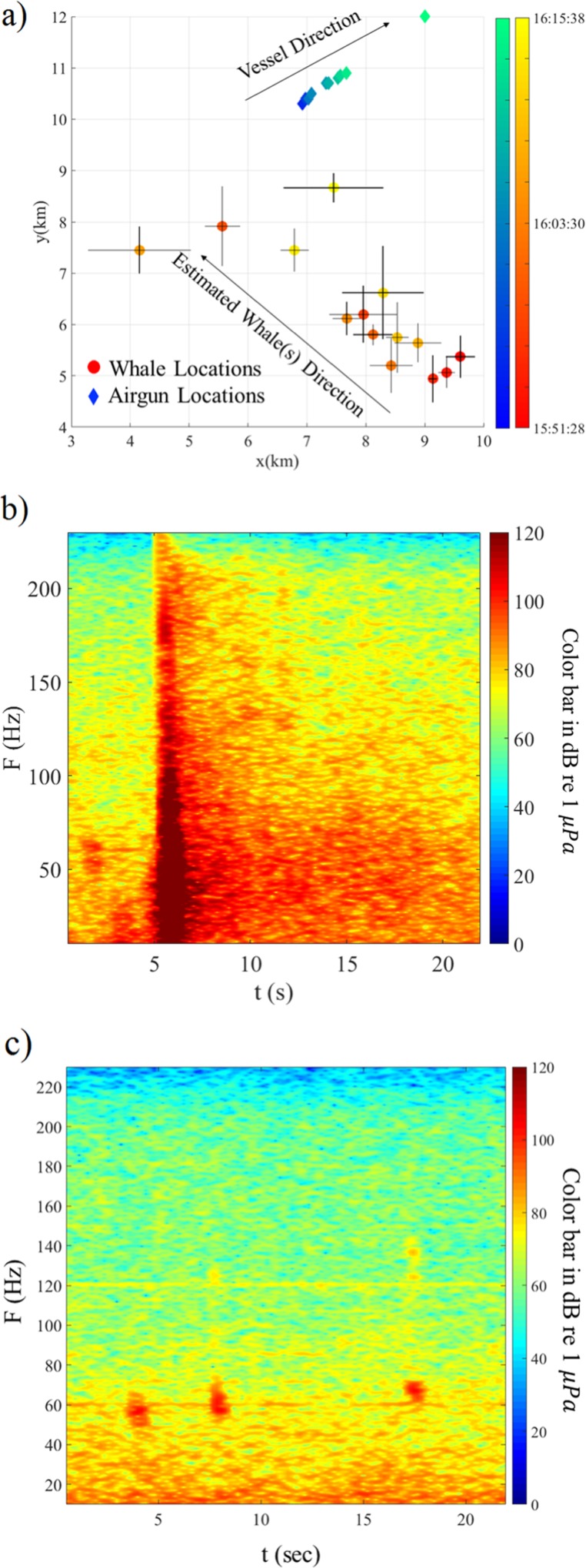
Location results on July 27, 2011. (a) Spatial distribution in Universal Transverse Mercator (UTM) coordinates of the calls recorded on July 27, 2011 relative to a reference point located in zone 5 at (-44, 6372) km. This figure has the same format as Fig 7A. (b) Spectrogram, in units of decibels re 1 μ*Pa*, of three whale calls (visible at 2 s at around 60 Hz) recorded on July 27, 2011 at 15:50 UTC concurrent with a full power airgun shot, (c) Spectrogram, in units of decibels re 1 μ*Pa*, of three whale calls (visible at 4 s, 8 s and 18 s at around 60 Hz) recorded on July 27, 2011 at 15:51 UTC when the airgun array was in a transient state to mitigation gun which started at 15:52 UTC.

The first whale call was detected by a couple of the elements on the streamers at 15:50 while all airguns were firing. This is a unique recording since the full airgun array and whale calls were recorded simultaneously (Fig 8B). Unfortunately, calculating a location was not possible because the SNR is very low due to reverberations from the full airgun array. After that shot, the airgun was powered down to the mitigation gun and another 14 whale calls were recorded up until 16:15 when the ramp-up procedure started, after which no whale calls were acoustically detected. These whale calls had different spectrograms and their frequencies varied between 40–100 Hz. One example is shown in Fig 8B. The water depth varied from 237 m to 222 m.

The spatial distribution of all the recorded calls (Fig 8A) show that the vocalizations traveled ~5 km in a northwest direction while the ship moved in a northeast direction. There is not enough information to indicate whether the recorded calls are coming from an individual whale or several whales but the localization are broadly consistent with the positions reported by the observers. There is no indication that the acoustically active animals either follow the mitigation gun or suddenly change their direction.

### 3. Detection event 3

The third detection event (Table 6) occurred on July 29, 2011 and comprised four groups of whales observed one after another. Each group approached the vessel close enough to trigger mitigation procedures. First a pod of unidentified baleen whales crossed ahead of the vessel, initiating a power-down of the source at 01:25. Just as these animals were determined to have departed the exposure radius, a pair of fin whales was sighted approaching the vessel, traveling antiparallel to the vessel and dropping astern and outside the exposure radius at 02:43. As the fin whales were being observed, a pair of humpback whales were sighted, one off the bow and one off the stern, blowing, fluking and breaching, all within the exposure zone. Later that afternoon, 4 humpback whales were observed at 05:06 travelling antiparallel to the vessel, diving and fluking.

**Table 6:** Summary of the acoustic and visual detections on July 29, 2011.

*Time of the important events*	*Information from the acoustic recordings*	*Information from the visual observation*	*Connecting the visual and acoustic monitoring*
1:25	Airgun array is firing at full power and the streamer data are too noisy to detect any call. Airgun array is powered down to the mitigation gun at 1:44.	2 unidentified baleen whales are observed blowing off the port bow. 2 additional blows are also sighted more than 4 km off the port bow.	Streamer data are too noisy. No connection is found.
2:15	Mitigation gun is on. No call is recorded.	Two close animals are sighted crossing ahead of the vessel at a moderate to fast pace. They are last sighted crossing ahead traveling southeast off the starboard bow.	No connection between the visual observation and the acoustic recordings.
2:43	Mitigation gun is on. No call is recorded.	Two fin whales are blowing off the starboard bow travelling antiparallel to the vessel. At the same time, two humpback whales are sighted one off the port stern and one off the port bow.	No connection between the visual observations and the acoustic recordings.
2:49	Mitigation gun is still on. One call is detected on the streamer. The estimated location is 6.2 km off the starboard bow.	The fin whales are sighted 1 km from the airgun at 2:58. The two humpback whales are sighted outside the exposure zone until 3:17.	Based on the visual observation, the recorded call is believed to be a humpback whale call.
4:22	Ramp-up is started at 4:15 and is completed at 4:23. The streamer data are too noisy to detect any call.	The humpback off the port bow is swimming parallel to the vessel but at a slower pace such that it drops along the port side and then astern. The humpback off the port stern is last sighted fluking and moving away from the vessel heading southwest.	Streamer data are too noisy. No connection is found.
5:06	The airgun array is powered down to the mitigation gun. Calls are recorded by the streamer. The estimated locations vary from 8 km to 14.4 km off both port and starboard sides. The ramp up is started at 5:36.	Two humpbacks are observed off starboard bow. Two more humpback whales are sighted blowing ~4 km off the port at 5:09.	The visual observations confirm the acoustic findings that there are whales on both sides of the vessel.

**Fig 9:**
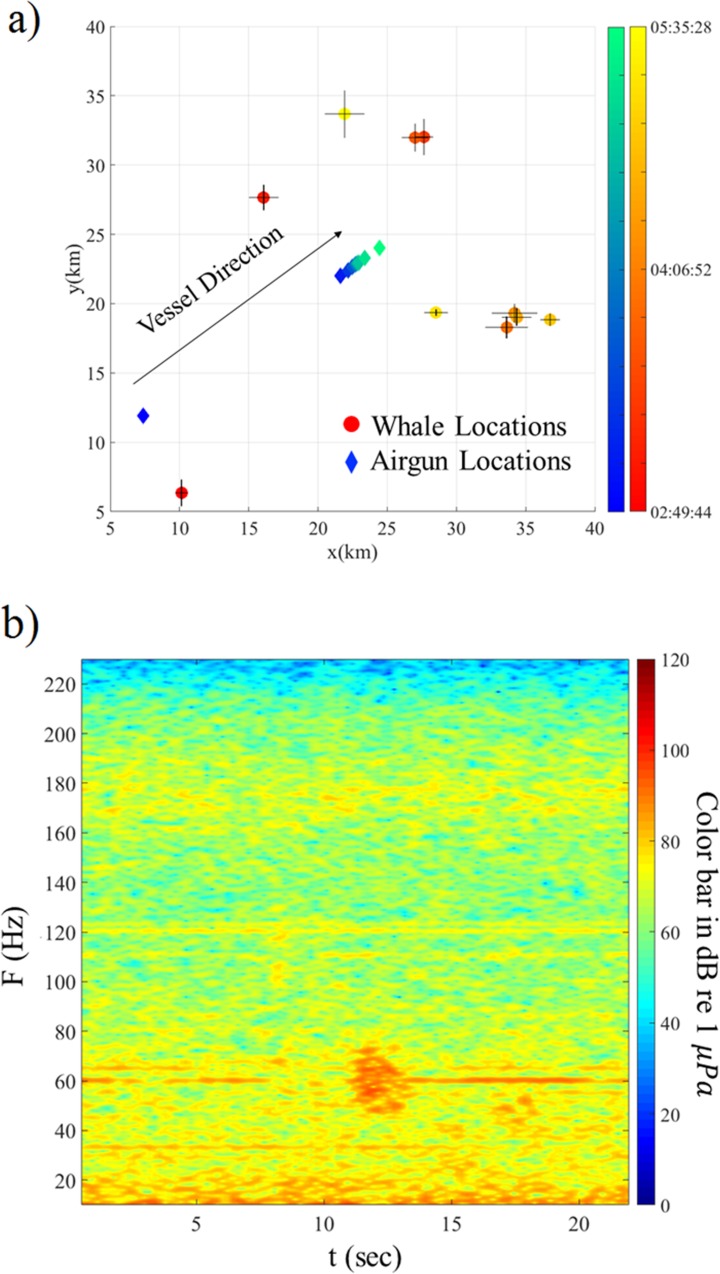
Location results on July 29, 2011. (a) Spatial distribution in Universal Transverse Mercator (UTM) coordinates of the calls recorded on July 29, 2011 relative to a reference point located in zone 5 at (25, 6240) km. This figure has the same format as Fig 7A. (b) Spectrogram in units of decibels re 1 μ*Pa* of a whale call (visible at 12 s and around 60 Hz) recorded on July 29, 2011 at 5:35. The mitigation gun is off for a moment in order to start the ramp up at 5:36.

The first whale call detected by the streamers occurred at 02:49 while the mitigation gun was being fired. No further whale calls were detected on the streamer data until 05:06 when another 8 calls were recorded. All the whale calls were at frequencies between 40–100 Hz (Fig 9A) and the water depth varied from 90 m to 93 m.

The spatial distribution of all the recorded calls (Fig 9A) shows that the estimated location of the first whale call at 2:49 is 6.2 km off the starboard side of the vessel. No clear temporal pattern is apparent in calls recorded between 05:06 and 05:35. Both the estimated locations and visual observations (Table 6) show that there were whales on both sides of vessel.

### 4. Detection event 4

The fourth detection event (Table 7) occurred on July 31, 2011 and involved a sighting of 6 humpback whales diving and fluking away from the vessel at 18:35 followed by the detection of a humpback whale swimming toward the vessel at 19:09. Visibility decreased due to encroaching fog starting at 19:11, but the humpback whale was observed again at 19:57, 1.3 km from the airguns. The ramp-up procedure after the first sighting was delayed due to the second sighting.

**Table 7:** Summary of the acoustic and visual detections on July 31, 2011.

*Time of the important events*	*Information from the acoustic recordings*	*Information from the visual observation*	*Connecting the visual and acoustic monitoring*
18:35	The airgun array is powered down to the mitigation gun. The first whale call is detected on the streamer. The estimated location is 2.0 km from the airguns in the starboard quarter.	One humpback whale is observed 1.76 km from the airguns moving away off the starboard bow. Also, five humpback whales are observed aft of the vessel well outside the exposure zone.	The visual observations confirm the acoustic results.
18:47	One call is recorded at 18:44 and the estimated range is 4.7 km. The ramp-up begins at 18:57.	The animal that was close to the airguns leaves the exposure zone. The visibility is reduced due to the encroaching fog started at 19:11.	The visual observations confirm the acoustic results.
19:59	The ramp-up is finished at 19:34. The airgun array is powered down again at 19:49. The last whale call is detected on the streamer. The estimated location is 13.0 km from the airguns in the starboard quarter.	One humpback whale is observed at 19:57, 1.3 km from the airguns off the bow traveling perpendicular to the vessel.	The visual observations at this time doesn’t match with the acoustic result. The acoustic recordings at this time may belong to the pod observed at 18:35 which based on the visual observations should be well out of the exposure zone by this time.

**Fig 10:**
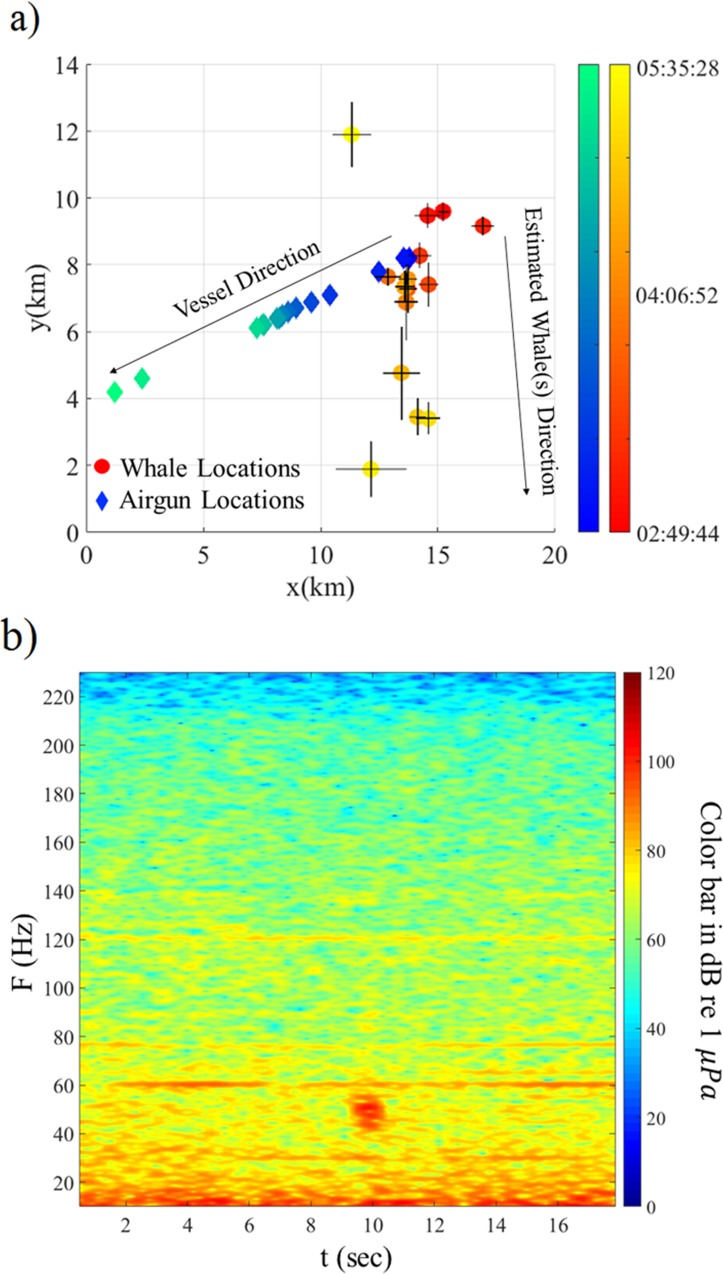
Location results on July 31, 2011. (a) Spatial distribution in Universal Transverse Mercator (UTM) coordinates of the calls recorded on July 31, 2011 relative to a reference point located in zone 5 at (-165, 6160) km. This figure has the same format as Fig 7A. (b) Spectrogram in units of decibels (dB) re 1 μ*Pa* of a whale call (visible at 10 s and around 50 Hz) recorded on July 31, 2011 at 18:35.

A total of 15 whale calls were recorded by the streamers between 18:35 and 19:59 in the frequency band 30–80 Hz (Fig 10B). The water depth varied from 95 m to 85 m. For this event, only data from the first streamer were used for locations (Fig 10A) since navigation data for the second streamer is not available due to technical problems that day.

The start of the ramp-up process coincided with the 4^th^ recorded call at 18:57. The distance between the estimated location and the airgun location increased from ~2 km to ~13 km as the animal(s) moved at a speed of ~2 m/s in a southerly direction while the vessel moved toward the southwest. Similar to the first and second events, there is no evidence that the vocally active animals either follow the mitigation gun or suddenly change their direction.

## VI. Discussion

More than 50 baleen whale calls are localized using dual seismic streamers during a seismic reflection survey in Alaska. In general, there is not enough information documented by PSOs to examine the accuracy of the acoustic results. Implications of the observations are discussed below, along with areas that require more supporting information or further analysis for verification:

1. Many baleen whale calls are detected on the streamer data, none of which are recorded by the short PAM array, deployed separately for marine mammal monitoring. Since streamers are wide aperture arrays and are designed to focus on low frequency signals, they appear to be more reliable tools for baleen whale monitoring compared with the short PAM array currently used by the R/V *Langseth* to monitor a wide range of animals (high frequency to low frequency vocalizations).
2. In this study, the whale vocalizations were detected on the streamer data in only 40% of the periods that the animals were observed by PSOs. This demonstrates a fundamental limitation of this method for mitigation, in that whales may often be silent. In this study, the visual observations are not sufficiently detailed to connect each acoustic detection to an observed animal even when acoustic and visual detections are nearly synchronous. However, the complementary nature of the two monitoring methods is evident.
3. This study shows how, because a streamer is rarely perfectly straight, the actual streamer locations help to resolve left-right ambiguity in locations. Although, the left-right ambiguity is not a significant problem in marine mammal monitoring applications (i.e. the distance from the airgun does not change regardless of side), knowing the exact location of the animals is important for studies of animal behavior during and after seismic surveys.
4. The airgun pulses usually cannot mask the whale calls since their regularity makes them easy to distinguish from a whale call. However, prolonged reverberation from the airgun pulses may mask the baleen whale calls. It has been documented that airgun activities increase the ambient noise level between pulses and reduce the PAM system performances in shallow-water seismic surveys [28]. In addition to the masking effect, the airgun reverberation noise and the vessel noise increase the uncertainty of the location results. This can be seen in the uncertainties associated with the estimated locations in Figs 7–10. The calls from further whales have lower SNR and thus larger uncertainties. The impact of SNR on the estimated locations is explored in the simulation (Section IV) by adding white noise into the synthetic signals. However, it may be more realistic to model the airgun reverberation by directional noise. This analysis is beyond the scope of this paper and is left as future work.
5. None of the estimated localization of vocalizations are within the exposure zone. The minimum estimated range is 1.6 km and was obtained during the mitigation process and so is well outside of the exposure zone (see Table 1). Similar results were previously reported for bowhead whales [4] although our dataset is too small to attempt a statistical analysis to determine whether vocalizing whales avoid the exposure zone.
6. The approach described in this paper can be used to assess baleen whales’ behavior in response to airguns. In this study, there is no indication that the acoustically active animals either follow the mitigation gun or suddenly change their direction. This observation is limited to the vocalizing animals and obviously cannot be extended to the silent whales. In order to draw any conclusion regarding animals’ response to airguns, more statistical analysis with larger data sets, including more accurate visual observation documents, are required.

## VII. Conclusions

Dual streamer data are used to detect baleen whale calls during a seismic reflection survey in Alaska. The low frequency localization method developed in a previous study [20] was used to estimate the location of the recorded baleen whale calls during the mitigation process. The locations of ~60 whale calls were estimated in four days, all during the mitigation procedures. Overall, three main conclusions can be drawn from this study: 1) seismic streamers are more reliable tools than the current *R/V Langseth* PAM system for locating and monitoring vocalizing baleen whales in the vicinity of seismic operations; 2) a single seismic streamer can usually resolve left/right ambiguity because it is rarely perfectly straight, but dual streamers significantly improve the location results; and 3) the data set used in this study is not sufficiently large to be used to infer animal behavior study, although no striking behavioral changes are observed. However, streamer data have the potential to be used for evaluating mitigation processes and studying baleen whale responses to airguns.

Future work could be focused on extending this method for real time monitoring during seismic surveys. This would require having automated whale call detection and localization algorithms that work in full power shooting modes, and more extensive error analysis to better assess the location uncertainty in low SNR.
